# Reach-to-grasp movements in *Macaca fascicularis* monkeys: the Isochrony Principle at work

**DOI:** 10.3389/fpsyg.2013.00114

**Published:** 2013-03-08

**Authors:** Luisa Sartori, Andrea Camperio-Ciani, Maria Bulgheroni, Umberto Castiello

**Affiliations:** Dipartimento di Psicologia Generale, University of PaduaPadua, Italy

**Keywords:** *Macaca fascicularis*, reaching movements, motor activity, isochrony principle, kinematics

## Abstract

Humans show a spontaneous tendency to increase the velocity of their movements depending on the linear extent of their trajectory in order to keep execution time approximately constant. Termed the isochrony principle, this compensatory mechanism refers to the observation that the velocity of voluntary movements increases proportionally with their linear extension. Although there is a wealth of psychophysical data regarding isochrony in humans, there is none regarding non-human primates. The present study attempts to fill that gap by investigating reach-to-grasp movement kinematics in free-ranging macaques. Video footage of monkeys grasping objects located at different distances was analyzed frame-by-frame using digitalization techniques. The amplitude of arm peak velocity was found to be correlated with the distance to be covered, and total movement duration remained invariant although target distances varied. Like in humans, the “isochrony principle” seems to be operative as there is a gearing down/up of movement velocity that is proportional to the distance to be covered in order to allow for a relatively constant movement duration. Based on a centrally generated temporal template, this mode of motor programming could be functional in macaques given the high speed and great instability of posture and joint kinematics characterizing their actions. The data presented here take research in the field of comparative motor control a step forward as they are based on precise measurements of spontaneous grasping movements by animals living/acting in their natural environment.

## Introduction

A number of studies have addressed grasping behavior in monkeys and apes (e.g., Christel, 1993; Spinozzi et al., 2004; Pouydebat et al., 2006), but only a few have considered the macaque species (Pouydebat et al., 2006; Macfarlane and Graziano, 2009). To date, the majority of these observational studies focused on hand shaping rather than on the reach-to-grasp movement in its entirety. This issue has been tackled by some comparative kinematic studies on reach-to-grasp behavior in the human species, in macaques living in captive, non-natural conditions (Fogassi et al., 1991; Roy et al., 2000, 2002, 2006; Christel and Billard, 2002; Pouydebat et al., 2009; Sacrey et al., 2009; Jindrich et al., 2011), and in free-ranging macaques (Sartori et al., 2013).

A large part of the data from these studies indicates similarities in hand shaping across species (Fogassi et al., 1991; Roy et al., 2000, 2002, 2006; Christel and Billard, 2002; Sacrey et al., 2009; Sartori et al., 2013). More specifically, hand aperture appears to be scaled depending on the object's size (e.g., Fogassi et al., 1991; Roy et al., 2000; Sartori et al., 2013). Total movement time is affected by the size of the object to be grasped as reaching to grasp a small object takes longer than reaching to grasp a larger one (e.g., Fogassi et al., 1991; Roy et al., 2000; Sartori et al., 2013). When differences across types of grasping (i.e., precision vs. power grips) are considered (Fogassi et al., 1991; Sartori et al., 2013), peak wrist velocity is lower and the moment when the animal's fingers start to close around an object is anticipated, just as in humans, during precision (i.e., small objects) compared to power grip movements (e.g., Gentilucci et al., 1991; Castiello, 1996).

As far as differences are concerned, not all general features characterizing human action were also noted in the animals. Roy et al. (2000) reported that in monkeys the amplitude of arm peak velocity and the time of maximum grip aperture appeared to be similar regardless of the size of the object to be grasped. In humans, the amplitude of peak velocity is higher and the time of maximum grip aperture occurs later for larger compared to smaller objects (e.g., Gentilucci et al., 1991; Jakobson and Goodale, 1991). Other authors noted relevant kinematic irregularities in the velocity and acceleration profiles of arm movements with a greater instability of posture and joint kinematics in macaques compared to humans (Christel and Billard, 2002).

Although these studies seem to favor the hypothesis that macaques and humans share a number of kinematic features, important differences have been noted, and the debate continues to unfold. For instance, an issue that has yet to be investigated in macaques, from a kinematical perspective, relates to how movements are planned depending on the distance the arm should cover for grasping an object and how this compares to humans. In this respect, the isochrony principle states that the velocity of a movement is proportionally linked to its linear extension (or trajectory) so as to permit the execution time to be maintained approximately constant (Viviani and McCollum, 1983). It has been suggested that this principle links velocity to the amplitude of a movement plan. Reference to this type of temporal regularity in human motor behavior was first made in the literature more than a hundred years ago (Binet and Courtier, 1893) and it has been noted in a variety of well-rehearsed actions. Studies on writing movements, for instance, have shown that it takes the same time to write a letter or a word at different sizes, implying that there are proportional changes in velocity (Michel, 1971; Lacquaniti et al., 1983). This type of relationship between the linear extension of a movement and velocity appears to be a rather common feature pertaining not only to writing but also to a variety of actions such as typing (Viviani and Terzuolo, 1982), lifting weights (Gachoud et al., 1983), kicking activity in infants (Thelen and Fisher, 1983), and hand and arm movements (Freund and Budingen, 1978; Jeannerod, 1984). In sharp contrast to the wealth of psychophysical data about isochrony in humans (e.g., Freund and Budingen, 1978; Binet and Courtier, 1893), little is known about how this principle applies to motor programming in non-human primates.

The present study was undertaken with the intent of ascertaining if macaque monkeys apply the isochrony principle as they execute actions they routinely carry out daily: grasping objects.

The main question is whether macaques gear down/up movement velocity depending on the distance from the target to be grasped to maintain a relatively constant movement duration.

Investigating isochrony in the arena of prehensile actions was considered the most favorable condition for testing the principle in comparative terms given that macaques naturally reach for and grasp objects very quickly, and the principle appears to be particularly true with reference to fast human arm and hand actions (Freund and Budingen, 1978). In the light of these observations, the following is a report on a systematic kinematic study on macaque monkeys living in totally natural conditions as they grasp objects located at different distances from them.

## Materials and methods

### Study species

Twenty adult macaques (*Macaca fascicularis*), all belonging to a single free-ranging troop made up of 65 animals living in Pulau Besar, Langawi, Malesia, were studied. The subject pool included 9 males and 11 females with an estimated age no less than four years.

### Data collection

A total of 10 h of video footage was filmed between 10.00 and 14.00 h daily from November 2 to November 27, 2008. The video was filmed *ad libitum* using a digital video camera. In view of the difficulty of filming any particular monkey grasping an object for any length of time before it moved away or turned its back, *ad libitum* rather than all-occurrence sampling was considered the most appropriate method to assess their behavior in natural conditions (Altmann, 1974). The monkeys were all filmed standing or sitting on the ground as they grasped objects during normal daily behavior. Every effort was made to avoid contact with the animals and the video footage was consequently filmed from a distance using a zoom lens. All the objects that were gripped/grasped were naturally found in the environment and were not introduced by the experimenters.

### Grip classification

Grips were classified by areas of skin surface contact which was possible to determine by observing the video frame sequences. Two expert judges, unaware of the study rationale and blind to the experimental conditions, assessed all the recordings for each subject. Reliability between the two judges was very high (Cohen's κ = 0.91). The present study exclusively concerned precision grip movements that could be unambiguously identified as such and that were performed to handle objects located at different distances. A precision or a pinch grip involves the end of the thumb and the distal pad of the index finger for fine manipulation, in the case of macaques, of small objects such as seeds, soil fragments, or blades of grass. In natural environments, spontaneous movements do not necessarily fit into the classical precision grip category: at times three fingers are involved, at others various finger combinations are utilized often changing fluidly from one configuration to another. Consequently some grips carried out by the macaques during filming did not fit into the simple precision grip category. Our analysis was nevertheless confined to this type of grip because the majority of movements (80%) performed upon the same objects located at different distances were performed using this kind of prehensile action.

### Data analysis

**Figure 1:**
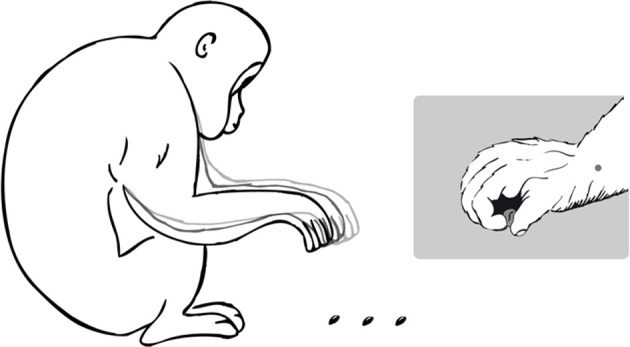
**Left Panel:** schematic drawing representing the posture adopted by the animal during the reach-to-grasp movement. Overlays indicate the movement performed at three different distances. **Right Panel**: positioning of the marker upon the wrist for the purpose of digitalization. Markers were located (*post-hoc*) on the wrist, and the distal phalanx of the thumb and index finger. A precision grip involving the tip of the forefinger and thumb to hold small objects is represented.

The video sample was analyzed frame-by-frame using an in-house software developed to perform two-dimensional (2D) *post-hoc* kinematical analysis (Castiello et al., 2010; see section “Data Analysis”). Care was taken to compare only those movements that were carried out while the animals were in a sitting position (i.e., with the elbow flexed and the torso bent forward). That position (Figure 1) was chosen because it facilitated comparison across kinematic studies on humans (e.g., Gentilucci et al., 1991) and macaques (e.g., Fogassi et al., 1991; Roy et al., 2000; Christel and Billard, 2002; Sartori et al., 2013). To avoid any skewing effect, only reaching movements performed along a plane perpendicular to the camera axis were analyzed. A frame of reference identifying X and Y axes as horizontal (ground) and vertical directions was manually set by an operator. A known length, selected case by case, in the camera's field of view and in the same plane as the movement was used as the measurement reference unit. As shown in Figure 1, a marker was then made on each subject's wrist to indicate the reaching component as a function of time. A marker positioned on the wrist is classically used to measure kinematics of the reaching component of a reach-to-grasp action in both monkeys Fogassi et al., 1991; Roy et al., 2000; Sartori et al., 2013 and humans (Gentilucci et al., 1991; Castiello, 1996). The starting position was defined as the right hand resting on the ground in between the legs. The hand starting area for the selected movements was similar across subjects (±0.3 cm^2^). Movement tracking procedures were then performed in order to extract a number of kinematic parameters based on spatial and temporal indexes. The following dependent variables, specifically tailored to test for specific isochrony effects were thus considered: (1) the total movement duration from the time the subject started the action to when its hand grasped the object (the criteria for determining movement initiation was zero wrist velocity); (2) the time of peak wrist velocity, defined as the time it took subjects to achieve maximum speed as they reached for the target; and (3) the amplitude of peak wrist velocity, defined as the maximum speed achieved by subjects as they reached for the target. Only grasping movements of the right hand directed toward one particular type of object (i.e., small balls of clay ~0.7 cm) located at three distances (12, 14, and 18 with an interval error of ±0.3, ±0.2, ±0.3 cm, respectively) were considered. All of the objects that were assessed were indigenous to that area and were not introduced into the environment by the experimenters. In accordance with the experimental protocol, the laterality quotient (LQ) was 75 (±12) with a LQ of 100 referring to a full right hand preference. Food items were not taken into consideration by this study because monkeys typically do not pause to grasp those objects but perform continuous joint movements as they reach for and take food to their mouths. For each of the subjects studied fifty movements of each of the three distances considered were chosen randomly from a larger sample. A repeated measures analysis of variance (ANOVA) on average values for each subject was carried out to compare object distances (12, 14, and 18 cm) for each dependent measure. Pearson's r correlation coefficients on mean values were calculated on the relationship between mean peak wrist velocity and distance from the target. Values of peak velocity were normalized to the highest value for each subject.

## Results

**Figure 2:**
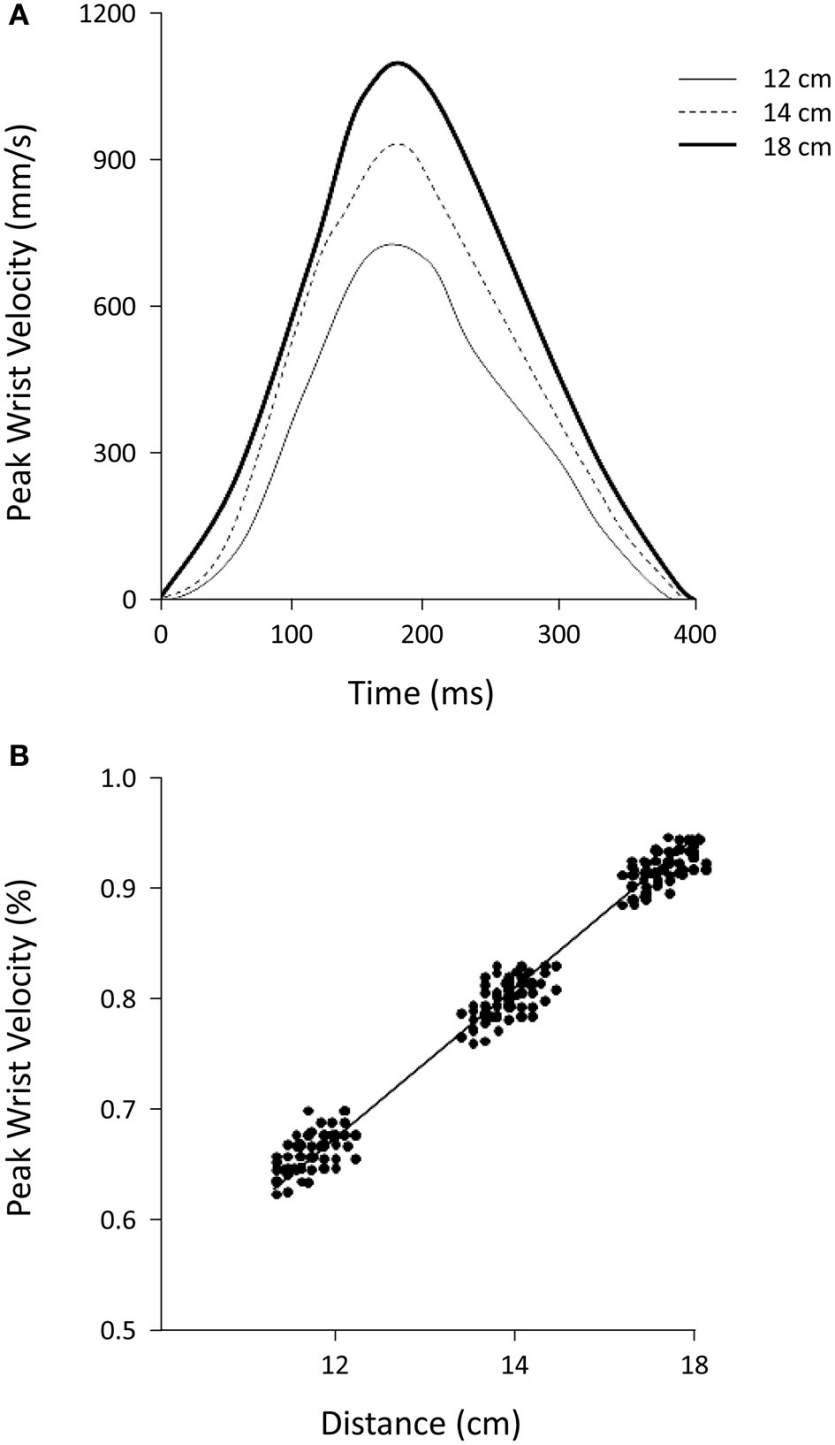
**(A)** Shows the average peak wrist velocity for objects located at different distances. **(B)** Shows the relationship between mean peak wrist velocity and distance from the target. Values of peak velocity were normalized to the highest value for each subject. A linear regression very accurately fits the data points. The data outlined in the two panels are from one representative subject (*N* = 8).

The total duration of the grasping movements toward the targets at the three distances studied did not vary [402 ± 6, 394 ± 6, and 400 ± 18 ms for 12, 14, and 18 cm, respectively; *F*_(2, 38)_ = 1.87, *P* = 0.17, η^2^_*p*_ = 0.09; Figure 2A]. The time of peak wrist velocity did not differ significantly with reference to the three distances studied [214 ± 4, 215 ± 7, and 212 ± 4 ms for 12, 14, and 18 cm, respectively; *F*_(2, 38)_ = 2.52, *P* = 0.09, η^2^_*p*_ = 0.12; Figure 2A]. The peak velocity amplitude was higher for movements performed to seize objects located 18 cm away with respect to those located 14 and 12 cm away [1201 ± 83, 986 ± 47, 876 ± 62 mm/s, respectively; *F*_(2, 38)_ = 216.09, *P* = 0.000, η^2^_*p*_ = 0.92; Figure 2A]. There were high correlations (*rs* = 0.90) between distances and peak velocities (Figure 2B) and lower correlations (*rs* = 0.23) between distances and movement durations. This pattern was found to be true for all of the subjects studied.

## Discussion

Due to the difficulty in carrying out systematic studies in unconstrained conditions, little is known about how non-human primates organize natural reach-to-grasp actions. Using the experimental protocol outlined here, it was possible to examine the animals' natural behavior in their normal habitat utilizing an experimental paradigm (*post-hoc* digitalization) to investigate freely performed movements by a large number of exemplars handling indigenous objects.

Focusing on whether macaque monkeys respect the isochrony principle as they perform reach-to-grasp actions to seize objects located at different distances, this study found that the time needed to execute an action remains constant, while the velocity is proportional to the distance to be covered. A high correlation was found between the distance to be covered and the peak velocity and a low one was found between the distance to be covered and movement duration. Given these results, we can conclude that the isochrony principle is at work. Like humans, macaques seem to be able to implement a proportional gearing up/down of movement velocity in connection to the distance to be covered. Studies on hand movements in humans have shown that the duration remains approximately constant for fast voluntary movements of hand/arm muscles regardless of their amplitude (Freund and Budingen, 1978). As the principle of isochrony seems to be at work in humans particularly when fast actions are being programmed, it is within this arena that any comparison across species in terms of isochrony should be made.

Macaques seem to naturally reach for objects very quickly and in any case quicker than humans (Christel and Billard, 2002). Faster movements are achieved by faster rotation and higher angular velocity and acceleration of the wrist. Macaques also show smaller shoulder abduction and wider elbow excursion throughout pronation together with larger movements of the torso (Christel and Billard, 2002). Despite their speed and greater instability of posture and joint kinematics, macaques show a hand choreography which is similarly smooth to that achieved by humans at a lower speed and mobilizing fewer joints. This is possible, we surmise, in virtue of the principle of isochrony, which appears to constitute a basic property of macaques' motor organization. Therefore this general compensatory mechanism seems to characterize macaques' motor acts. They proportionally tie the velocity of a movement to its linear extension so that the execution time is maintained approximately constant. This might indicate that macaques tend to link velocity to the amplitude of a movement plan.

This type of programming keeps the timing of the commands independent from the spatial parameters of the movement. In other words, selection of the muscles needing to be activated to carry out a given task can be modified, or the torque applied to the joints can be modulated within a centrally generated temporal template that determines the co-ordination of a given action. This appears to be the easiest and most readily chosen organizational option of the neural system to compensate for the postural and joint kinematic instability characterizing macaques' reach-to-grasp actions.

## Conclusions

Despite the difficulties encountered in conducting this research project, these findings provide new information delineating how macaques' reach-to-grasp behavior naturally unfolds. The study, in fact, adds a novel finding in the literature by delineating that isochrony constitutes a very basic property of macaques' motor organization as noticed in humans for certain tasks.

This study presents some limitations. The first is that it utilized two- rather than three-dimensional kinematics, but a two-dimensional approach is the only way to film movements in totally natural, unconstrained conditions. A great amount of energy was dedicated to establishing the experimental criteria of the movements to be analyzed (see section “Materials and Methods”). The second limitation is that the work does not present a full report on homologies across species. Our analyses were confined to macaques and possible parallelisms with humans grasping actions. Debating if the isochrony principle apparently driving reach-to-grasp behavior in macaques is shared by many animals who perform similar actions (e.g., Iwaniuk and Whishaw, 2000; Sacrey et al., 2009) fell outside the scope of the present study. The third limitation is that our analysis focused exclusively on precision grips and did not consider a wider range of grip behaviors, objects, and postures. These factors should be considered in future research concerned with the very nature of the development and the mechanisms underlying the emergence of this specific characteristic of macaques motor behavior.

### Conflict of interest statement

The authors declare that the research was conducted in the absence of any commercial or financial relationships that could be construed as a potential conflict of interest.
