# Supplementary material for: Reach-to-grasp movements in Macaca fascicularis monkeys: the Isochrony Principle at work
Source: Front Psychol. 2013 Mar 8;4:114. doi: 10.3389/fpsyg.2013.00114 (PMC3592261; doi:10.3389/fpsyg.2013.00114)
Supplement: Supplementary file 1 [file DataSheet1.PDF]

Monkey	Trial	Movement Duration			Time to Peak Velocity			Amplitude Peak Velocity		
		12 cm	14 cm	18 cm	12 cm	14 cm	18 cm	12 cm	14 cm	18 cm
1	1	398	378	422	219	217	209	885	988	1232
1	2	358	401	385	217	214	220	901	1001	1288
1	3	453	398	401	212	219	209	888	996	1268
1	4	432	411	396	212	214	200	714	985	1321
1	5	412	404	385	200	221	214	865	986	1298
1	6	404	398	401	215	210	200	876	932	1245
1	7	376	389	395	221	218	196	888	978	1278
1	8	412	376	385	212	216	179	856	999	1245
1	9	401	400	387	224	222	217	784	986	1225
1	10	384	387	395	196	210	198	861	1012	1126
1	11	391	422	385	234	210	214	845	1015	1258
1	12	414	387	378	222	231	215	863	965	1200
1	13	400	432	374	202	209	212	842	1078	1301
1	14	400	398	400	212	218	200	874	901	1325
1	15	386	356	385	209	215	202	745	965	1256
1	16	421	412	389	210	208	214	905	985	1241
1	17	399	388	385	208	216	201	956	997	1289
1	18	385	345	345	213	215	198	975	1002	1245
1	19	377	415	382	217	214	191	962	1005	1245
1	20	423	421	389	222	215	209	924	1015	1324
1	21	461	456	347	206	215	201	976	986	1329
1	22	458	436	375	231	216	211	924	958	1378
1	23	354	387	369	216	222	219	936	963	1387
1	24	387	376	358	205	220	198	912	954	1356
1	25	402	388	345	214	215	209	965	985	1345
1	26	412	396	346	222	206	216	898	966	1258
1	27	436	423	402	210	208	200	905	978	1328
1	28	444	435	399	207	210	201	845	992	1226
1	29	355	367	385	204	206	217	888	978	1258
1	30	348	354	386	208	214	221	965	1000	1296
1	31	401	432	374	203	217	209	915	1045	1254
1	32	388	387	369	212	217	212	958	1089	1247
1	33	376	356	400	217	215	209	975	987	1333
1	34	402	402	398	216	216	213	969	1025	1254
1	35	412	401	402	215	219	216	805	963	1289
1	36	389	378	411	217	189	209	872	989	1296
1	37	422	398	398	218	196	211	927	1000	1267
1	38	417	423	365	221	214	218	946	1002	1285
1	39	400	378	345	218	222	215	989	1006	1276
1	40	387	385	389	212	215	209	935	984	1202
1	41	392	386	379	205	209	212	974	1006	1300
1	42	389	401	367	206	212	209	965	1008	1245
1	43	401	399	395	202	212	215	987	1012	1265
1	44	396	389	345	201	208	214	962	965	1208
1	45	389	378	368	200	204	201	945	989	1205
1	46	400	387	367	210	219	204	856	1006	1321
1	47	398	400	356	211	216	213	906	963	1327

1	48	403	391	423	208	219	231	945	1014	1289
1	49	389	378	358	214	220	217	888	1061	1256
1	50	400	421	349	219	215	213	975	901	1325
2	1	367	358	378	209	198	215	896	991	1125
2	2	401	387	396	213	215	219	886	996	1196
2	3	349	402	385	212	216	213	898	1002	1145
2	4	421	387	397	222	214	219	924	1005	1178
2	5	389	401	396	219	175	218	786	1012	1196
2	6	400	388	359	221	196	199	856	987	1204
2	7	378	376	389	217	215	213	802	963	1278
2	8	454	438	378	221	222	215	865	893	1056
2	9	422	399	391	206	236	212	854	956	1189
2	10	396	402	392	208	223	202	825	845	1145
2	11	401	388	358	217	218	215	846	965	1176
2	12	400	378	366	210	219	214	832	987	1132
2	13	376	387	399	210	215	198	871	999	1156
2	14	385	401	401	210	217	200	863	1025	1205
2	15	398	377	395	219	225	217	954	1068	1209
2	16	388	387	402	213	219	200	836	985	1211
2	17	379	412	413	216	222	212	854	974	1198
2	18	398	400	396	214	214	212	854	963	1189
2	19	401	387	387	214	231	218	821	1011	1178
2	20	399	400	402	218	212	200	798	1025	1168
2	21	422	412	431	216	218	228	785	1089	1205
2	22	401	398	365	220	225	199	891	965	1247
2	23	431	422	398	210	216	212	814	932	1269
2	24	444	395	378	211	205	211	888	954	1196
2	25	376	392	345	215	218	209	906	987	1136
2	26	402	399	387	214	209	212	881	963	1258
2	27	389	400	385	218	212	200	805	987	1302
2	28	402	420	386	235	227	226	867	993	1269
2	29	421	387	394	200	219	198	812	1014	1215
2	30	389	377	388	218	225	216	799	1012	1198
2	31	400	396	401	212	216	198	824	996	1163
2	32	432	421	399	221	221	198	890	998	1154
2	33	401	421	418	214	227	214	802	859	1147
2	34	444	431	422	216	235	218	831	900	1156
2	35	423	418	421	220	218	215	802	932	1189
2	36	387	398	399	225	228	209	888	1014	1206
2	37	432	404	408	212	224	211	854	1006	1147
2	38	389	376	385	214	218	198	804	1008	1136
2	39	423	411	406	214	241	201	815	1047	1247
2	40	401	388	396	214	230	212	845	986	1196
2	41	377	387	387	218	218	206	952	996	1098
2	42	423	412	402	220	231	200	854	1005	1069
2	43	400	387	399	216	241	195	869	986	1145
2	44	412	406	415	221	218	220	812	999	1163
2	45	394	387	418	217	219	214	854	1012	1198
2	46	387	387	403	215	221	200	932	986	1025
2	47	401	401	409	217	219	205	845	987	1209

2	48	454	461	419	219	234	216	902	982	1163
2	49	423	411	386	215	241	218	921	1013	1278
2	50	400	385	395	222	222	217	966	1000	1026
3	1	378	421	385	216	206	205	808	988	1189
3	2	387	398	387	217	221	207	845	956	1156
3	3	387	401	395	210	209	211	862	987	1145
3	4	432	431	365	200	195	201	845	921	1143
3	5	395	400	378	206	189	197	902	1025	1125
3	6	421	421	389	218	227	199	812	1014	1254
3	7	445	411	374	217	236	221	912	1063	1296
3	8	398	400	392	219	215	202	926	996	1154
3	9	387	378	391	217	218	190	892	956	1278
3	10	399	389	365	221	224	213	789	987	1265
3	11	403	411	398	219	209	198	763	986	1325
3	12	384	398	378	211	215	207	805	999	1096
3	13	388	391	354	206	222	214	856	1021	1158
3	14	359	376	366	217	209	211	921	1014	1169
3	15	400	378	378	214	224	216	837	1036	1145
3	16	386	385	388	221	215	219	863	1000	1168
3	17	402	412	376	229	224	216	854	987	1149
3	18	434	422	345	213	219	221	872	986	1158
3	19	441	410	399	212	239	200	965	999	1146
3	20	387	376	356	214	215	182	885	987	1178
3	21	398	399	377	215	210	208	963	965	1211
3	22	422	402	399	200	241	196	954	921	1198
3	23	388	378	406	207	226	205	967	1000	1185
3	24	401	400	411	219	219	205	981	1004	1205
3	25	396	417	418	217	218	223	745	993	1196
3	26	421	403	409	216	247	205	888	992	1236
3	27	402	401	399	214	231	200	854	978	1149
3	28	388	389	385	218	224	199	831	945	1250
3	29	399	400	384	210	219	215	926	1056	1247
3	30	387	391	377	211	209	198	931	965	1198
3	31	400	393	369	214	210	190	854	989	1045
3	32	412	409	388	214	225	200	902	999	1163
3	33	425	403	366	237	229	201	788	1014	1223
3	34	398	387	375	219	215	209	754	1032	1247
3	35	388	385	382	202	209	197	904	896	1213
3	36	405	400	381	212	219	201	922	956	1147
3	37	435	421	475	218	238	196	934	965	1268
3	38	422	414	406	220	235	203	912	987	1196
3	39	411	403	402	214	221	200	936	988	1201
3	40	427	412	395	216	218	200	856	982	1096
3	41	398	422	441	218	211	236	996	1010	1214
3	42	400	398	396	223	231	200	875	963	1198
3	43	403	401	385	218	222	201	904	987	1187
3	44	391	388	365	222	210	196	985	1006	1186
3	45	400	392	388	228	209	195	925	987	1298
3	46	387	388	391	222	215	211	914	1054	1325
3	47	404	400	385	231	222	196	963	1056	1247

3	48	400	398	402	227	215	199	869	1000	1012
3	49	400	368	354	231	226	210	865	963	1125
3	50	377	369	378	227	198	191	802	987	1204
4	1	406	387	375	200	218	213	954	1000	1106
4	2	375	365	386	216	224	215	945	988	1232
4	3	432	428	391	215	235	216	896	965	1221
4	4	445	438	365	217	241	221	863	987	1209
4	5	456	476	385	215	248	198	984	1052	1231
4	6	387	401	374	216	215	208	953	1089	1185
4	7	405	421	356	214	220	215	965	1012	1191
4	8	436	451	378	212	231	198	945	1000	1205
4	9	447	400	377	214	229	206	978	1005	1187
4	10	398	356	385	215	216	209	856	1002	1154
4	11	421	404	381	223	225	207	878	985	1163
4	12	432	412	386	241	241	211	962	954	1222
4	13	412	417	359	234	221	201	946	947	1145
4	14	422	411	367	225	228	217	923	964	1163
4	15	406	406	365	224	215	214	865	1015	1198
4	16	401	398	391	231	206	213	789	985	1156
4	17	400	402	349	241	215	212	902	999	1188
4	18	400	399	406	213	212	202	876	1078	1178
4	19	359	387	422	216	198	218	892	985	1201
4	20	362	377	411	218	205	210	925	954	1265
4	21	346	395	418	226	207	212	938	1045	1156
4	22	376	394	398	224	213	199	961	954	1189
4	23	423	418	401	218	212	198	954	987	1204
4	24	396	387	389	210	222	205	902	985	1206
4	25	422	423	406	238	230	200	945	932	1189
4	26	417	405	399	225	218	213	916	1002	1152
4	27	432	428	402	231	235	225	876	987	1147
4	28	399	387	412	222	224	211	815	1000	1169
4	29	407	394	458	225	218	236	809	956	1138
4	30	428	411	365	210	222	208	922	987	1240
4	31	451	444	328	267	231	226	845	1012	1268
4	32	434	412	400	245	229	198	876	1021	1257
4	33	454	400	401	227	232	197	823	989	1196
4	34	421	398	432	231	241	231	888	997	1258
4	35	432	397	395	216	233	198	869	1001	1204
4	36	444	399	354	231	239	199	905	1078	1288
4	37	398	402	387	219	218	198	865	1100	1206
4	38	401	387	365	221	224	218	879	954	1352
4	39	422	412	406	209	216	205	863	985	1214
4	40	451	400	398	254	236	221	896	963	1089
4	41	432	412	425	211	225	218	854	954	1163
4	42	444	423	388	243	231	218	798	976	1157
4	43	387	396	356	218	210	205	897	923	1059
4	44	404	400	367	216	200	219	863	1054	1204
4	45	453	401	389	238	225	217	956	954	1211
4	46	437	423	345	229	224	211	902	963	1258
4	47	404	411	402	221	219	212	954	954	1267

4	48	387	405	355	215	209	209	932	965	1152
4	49	401	412	366	214	212	217	945	987	1145
4	50	336	398	345	221	189	215	802	1014	2147
5	1	368	387	389	218	212	201	856	984	1258
5	2	398	382	401	215	224	200	845	1012	1206
5	3	371	380	422	200	214	214	890	987	1207
5	4	421	402	368	221	235	218	836	986	1211
5	5	419	412	378	213	221	211	854	1012	1216
5	6	398	399	358	221	218	210	902	998	1396
5	7	402	401	400	231	229	198	912	956	1387
5	8	378	387	396	222	225	217	945	945	1387
5	9	412	410	419	210	210	201	903	978	1305
5	10	422	411	405	208	222	215	907	964	1274
5	11	416	422	414	223	234	206	889	1014	1258
5	12	409	398	400	219	219	198	875	986	1369
5	13	398	356	378	222	208	217	881	987	1423
5	14	406	412	391	214	219	201	735	1014	1263
5	15	388	393	385	225	221	198	902	1121	1258
5	16	407	402	354	221	231	214	854	954	1327
5	17	405	412	392	221	219	189	901	987	1395
5	18	399	400	368	218	231	217	912	963	1376
5	19	401	398	378	231	242	215	945	952	1204
5	20	376	378	391	221	198	189	925	947	1282
5	21	403	401	357	222	205	218	900	999	1456
5	22	412	412	402	226	197	200	852	1012	1399
5	23	401	399	400	228	206	217	865	1045	1322
5	24	421	412	401	227	210	199	883	985	1254
5	25	411	409	412	223	218	200	881	975	1289
5	26	389	400	398	218	204	201	845	973	1293
5	27	367	376	365	217	216	221	879	985	1226
5	28	345	338	378	225	219	181	902	968	1256
5	29	387	391	357	221	221	219	915	1000	1278
5	30	401	400	366	220	218	212	932	1016	1398
5	31	424	418	398	237	241	211	915	1001	1324
5	32	411	412	400	234	219	198	933	1008	1328
5	33	421	427	412	220	220	201	891	987	1258
5	34	388	406	387	211	217	191	782	961	1247
5	35	397	378	391	219	216	185	801	892	1346
5	36	388	391	358	206	228	208	902	965	1389
5	37	326	387	391	214	231	224	898	945	1356
5	38	325	345	365	203	218	205	874	1025	1442
5	39	400	399	400	207	226	198	856	1006	1365
5	40	412	401	358	210	234	204	784	965	1201
5	41	416	411	412	206	218	221	826	985	1389
5	42	419	400	365	219	219	186	854	974	1225
5	43	398	415	389	220	220	191	897	984	1201
5	44	398	408	378	218	245	186	863	998	1432
5	45	422	401	359	200	232	215	874	978	1201
5	46	387	400	365	216	215	211	862	1005	1458
5	47	401	388	364	229	221	217	891	945	1243

5	48	356	376	358	225	228	214	745	967	1345
5	49	367	358	391	209	219	200	711	981	1256
5	50	377	399	384	228	216	211	856	987	1245
6	1	354	376	381	212	222	198	886	994	1122
6	2	367	384	396	217	205	200	854	996	1201
6	3	402	398	354	221	219	213	832	974	1169
6	4	389	405	387	199	224	191	891	1012	1158
6	5	378	388	396	215	221	201	874	1056	1145
6	6	401	400	392	214	218	200	802	1047	1169
6	7	376	387	368	219	225	198	904	985	1158
6	8	381	398	346	222	213	208	865	988	1204
6	9	400	400	402	210	226	201	845	999	1165
6	10	354	355	399	211	218	200	832	1012	1169
6	11	378	371	356	215	224	214	854	1016	1145
6	12	398	401	368	201	228	221	812	954	1158
6	13	401	418	403	231	231	223	906	963	1147
6	14	399	402	369	209	210	205	907	954	1178
6	15	400	399	365	219	208	212	881	982	1196
6	16	400	406	345	226	215	196	886	963	1256
6	17	400	432	377	232	215	195	854	999	1198
6	18	376	389	374	210	217	201	856	995	1296
6	19	387	377	361	217	227	218	832	974	1125
6	20	357	356	395	200	215	213	821	956	1145
6	21	410	397	382	215	234	222	835	993	1189
6	22	421	413	367	211	219	217	892	976	1204
6	23	370	389	354	206	215	208	854	981	1269
6	24	411	413	412	213	209	206	800	983	1149
6	25	376	400	399	200	215	214	798	1012	1250
6	26	385	376	388	209	226	198	763	1062	1287
6	27	401	412	403	234	231	218	902	958	1198
6	28	398	399	422	214	215	221	825	962	1201
6	29	401	358	388	220	222	216	911	896	1275
6	30	354	359	403	212	199	215	806	991	1165
6	31	336	367	421	209	189	210	812	1026	1198
6	32	365	381	406	221	212	206	825	1014	1251
6	33	356	400	368	198	224	194	833	985	1145
6	34	362	387	396	201	189	213	878	962	1178
6	35	387	391	389	200	168	190	854	978	1169
6	36	391	400	394	219	199	201	862	994	1158
6	37	401	387	344	225	215	207	843	965	1145
6	38	414	413	409	246	225	210	856	982	1147
6	39	422	428	399	238	231	200	812	978	1123
6	40	398	400	409	200	234	215	821	912	1156
6	41	387	389	421	217	215	221	814	965	1148
6	42	389	398	399	201	239	206	832	984	1189
6	43	405	405	368	213	214	205	836	964	1256
6	44	356	387	395	197	208	200	822	971	1198
6	45	405	400	406	223	215	201	814	1016	1278
6	46	398	402	369	219	207	213	833	1009	1311
6	47	422	418	402	215	204	216	801	984	1289

6	48	395	396	409	222	199	218	812	972	1145
6	49	426	421	369	210	215	211	836	966	1179
6	50	398	402	366	200	225	192	821	987	1147
7	1	398	401	381	210	218	191	805	995	1225
7	2	359	367	385	216	221	189	891	965	1269
7	3	361	354	402	223	219	204	706	1025	1125
7	4	444	389	401	199	232	231	874	975	1249
7	5	398	391	389	212	215	215	863	965	1243
7	6	411	395	385	233	229	196	745	1056	1256
7	7	385	376	398	213	215	214	841	1024	1226
7	8	398	400	396	215	204	219	854	965	1235
7	9	400	388	387	221	236	224	852	945	1247
7	10	387	378	345	215	215	198	800	978	1256
7	11	396	395	365	218	228	201	821	991	1276
7	12	421	401	387	241	217	212	789	957	1265
7	13	399	411	373	208	209	195	841	986	1248
7	14	388	397	422	214	210	216	812	1026	1269
7	15	399	405	397	219	212	215	754	1049	1254
7	16	413	416	435	217	223	224	802	986	1257
7	17	400	408	402	202	219	213	831	1012	1248
7	18	383	396	413	209	222	212	803	954	1236
7	19	378	381	395	214	225	200	856	1000	1252
7	20	411	412	387	220	219	215	865	1036	1278
7	21	434	425	366	203	208	189	831	989	1241
7	22	408	395	365	217	215	193	852	954	1201
7	23	378	381	402	219	219	209	876	966	1165
7	24	394	388	412	220	221	227	963	982	1201
7	25	387	376	365	218	218	205	821	893	1299
7	26	402	412	400	211	225	198	874	1025	1163
7	27	435	434	398	216	231	206	956	1014	1204
7	28	422	417	377	216	237	200	889	989	1155
7	29	366	387	385	218	198	198	906	1012	1166
7	30	347	395	366	219	210	196	981	1046	1250
7	31	398	387	385	213	214	206	880	999	1144
7	32	397	367	374	225	219	214	896	985	1148
7	33	381	391	389	209	208	201	832	976	1166
7	34	422	403	365	214	231	211	854	988	1089
7	35	413	415	387	219	211	192	885	1015	1156
7	36	399	400	389	222	216	214	856	1002	1205
7	37	400	388	354	224	223	209	862	986	1219
7	38	414	376	369	200	218	191	878	991	1123
7	39	398	365	358	213	224	205	863	954	1165
7	40	356	385	401	222	227	215	866	965	1208
7	41	382	378	426	218	218	231	889	993	1125
7	42	387	400	399	220	206	198	865	956	1201
7	43	413	402	444	214	231	251	890	984	1089
7	44	410	409	422	231	218	224	856	952	1023
7	45	398	387	385	224	225	217	900	1063	1166
7	46	400	398	400	221	214	205	888	978	1025
7	47	376	356	368	222	210	199	862	989	1102

7	48	401	400	357	231	219	217	903	962	1204
7	49	388	381	395	224	210	202	876	996	1212
7	50	398	387	400	218	209	204	865	1015	1269
8	1	376	386	401	189	191	192	740	940	985
8	2	398	401	398	201	189	201	676	900	956
8	3	354	402	369	202	175	198	688	872	926
8	4	416	398	368	221	201	198	656	865	946
8	5	388	400	354	200	205	193	891	900	985
8	6	402	412	387	206	198	201	646	846	925
8	7	387	369	399	213	185	235	636	982	1250
8	8	435	417	388	234	196	214	674	885	954
8	9	412	411	356	215	184	193	601	878	963
8	10	387	387	399	222	205	219	645	891	954
8	11	410	399	400	218	211	225	700	874	921
8	12	387	403	409	208	205	231	787	862	987
8	13	384	444	451	212	214	258	752	890	932
8	14	383	398	335	217	231	216	674	874	905
8	15	400	354	368	216	198	194	602	856	986
8	16	391	387	395	202	199	215	665	841	963
8	17	382	376	375	214	205	216	645	852	984
8	18	398	400	412	219	201	205	679	871	978
8	19	400	402	432	220	215	200	632	881	956
8	20	389	398	387	217	214	196	688	885	932
8	21	412	401	411	210	213	209	625	889	988
8	22	418	417	416	215	198	225	641	885	945
8	23	411	422	426	219	178	231	765	841	977
8	24	412	417	429	200	185	222	689	851	956
8	25	387	388	392	188	198	198	674	886	936
8	26	399	401	396	219	205	192	691	885	945
8	27	376	387	394	222	196	201	675	854	985
8	28	388	391	388	176	207	199	665	822	965
8	29	402	345	356	216	205	217	695	885	925
8	30	391	394	406	220	205	221	689	845	981
8	31	400	388	421	221	205	222	621	885	895
8	32	428	416	418	209	198	214	645	852	924
8	33	411	397	400	187	211	199	621	845	985
8	34	423	401	396	213	183	185	665	806	954
8	35	417	387	378	217	187	212	645	845	985
8	36	388	392	405	221	186	213	632	812	986
8	37	413	422	406	233	191	214	691	816	895
8	38	400	399	369	227	193	199	600	821	888
8	39	413	421	389	218	211	191	692	821	856
8	40	399	378	378	219	203	218	664	812	965
8	41	387	393	401	216	198	196	636	845	921
8	42	402	388	399	211	201	200	654	874	924
8	43	388	401	412	209	222	211	671	832	953
8	44	411	412	431	222	189	218	688	821	987
8	45	388	396	368	219	158	196	695	889	925
8	46	386	391	389	216	197	192	692	865	945
8	47	421	412	403	207	209	209	654	904	958

8	48	434	424	435	231	187	235	665	802	936
8	49	433	411	387	234	211	222	645	854	958
8	50	399	400	394	218	187	189	619	841	963
9	1	365	371	387	205	210	201	954	988	1188
9	2	398	400	369	212	205	205	875	984	1156
9	3	400	378	356	225	216	201	892	978	1198
9	4	422	391	389	217	223	213	876	963	1178
9	5	387	388	406	219	215	221	885	1021	1205
9	6	411	402	389	206	221	198	845	1012	1241
9	7	435	421	365	208	242	212	865	1025	1126
9	8	387	397	398	210	215	209	891	965	1158
9	9	367	378	345	211	215	199	897	987	1205
9	10	376	381	378	209	221	215	879	945	1206
9	11	401	399	395	225	225	198	964	963	1198
9	12	389	387	391	214	228	211	882	977	1156
9	13	377	371	356	216	219	216	945	999	1145
9	14	400	394	345	213	231	221	893	1012	1204
9	15	400	381	395	218	219	217	874	1052	1258
9	16	356	402	392	222	189	211	800	1004	1265
9	17	412	421	402	218	234	209	954	985	1245
9	18	422	436	416	214	229	224	902	1002	1196
9	19	431	403	399	218	219	218	984	952	1178
9	20	385	398	354	222	216	208	903	1005	1156
9	21	404	414	369	218	222	200	954	1122	1206
9	22	412	402	358	215	225	201	862	985	1148
9	23	398	389	399	219	224	209	845	999	1169
9	24	400	400	402	225	228	221	878	996	1206
9	25	396	377	369	214	226	217	832	987	1254
9	26	431	422	409	209	216	261	865	954	1147
9	27	412	398	422	208	208	218	896	923	1169
9	28	399	400	421	214	224	219	924	915	1147
9	29	378	376	436	200	213	231	902	1026	1158
9	30	367	398	396	208	221	221	896	985	1169
9	31	399	402	421	201	205	218	956	945	1125
9	32	405	412	365	211	219	198	865	1012	1206
9	33	415	413	387	228	235	213	893	1026	1199
9	34	400	398	411	201	232	215	879	986	1204
9	35	384	388	402	210	215	212	866	996	1188
9	36	400	405	395	216	237	224	954	994	1165
9	37	412	422	368	213	235	215	891	992	1205
9	38	435	417	387	222	241	204	905	912	1198
9	39	421	387	368	218	211	218	925	987	1204
9	40	428	412	399	214	228	212	875	1015	1123
9	41	388	376	366	215	225	216	848	956	1056
9	42	396	385	354	224	210	218	894	996	1098
9	43	401	401	387	215	219	201	863	1025	1198
9	44	399	388	399	218	213	215	927	987	1205
9	45	387	376	406	216	236	215	863	1045	1154
9	46	388	382	419	210	217	224	877	982	1260
9	47	401	400	383	219	227	204	869	995	1198

9	48	400	387	391	215	214	196	915	1025	1157
9	49	345	356	365	217	189	201	874	1154	1204
9	50	415	403	388	228	221	218	896	935	1200
10	1	421	384	356	212	201	212	954	1012	1124
10	2	402	389	389	208	198	192	874	1054	1169
10	3	400	402	345	214	225	212	876	1026	1205
10	4	398	412	369	215	231	219	889	986	1189
10	5	376	356	402	209	225	212	893	995	1144
10	6	356	378	419	218	226	232	895	1036	1025
10	7	377	387	417	221	219	236	845	1012	1263
10	8	421	433	406	208	225	258	879	1002	1289
10	9	437	402	416	214	214	224	785	1047	1145
10	10	378	398	365	216	225	201	856	985	1204
10	11	427	423	389	200	215	212	788	974	1206
10	12	421	422	378	216	219	193	802	988	1189
10	13	400	400	395	214	254	201	835	999	1145
10	14	402	412	369	215	225	217	921	1025	1122
10	15	406	398	402	219	231	215	891	1144	1025
10	16	405	391	366	215	241	225	875	1021	1069
10	17	399	384	395	217	216	205	864	985	1250
10	18	387	401	425	213	209	231	876	965	1145
10	19	367	378	387	214	206	205	881	914	1205
10	20	389	388	391	219	215	200	902	1026	1169
10	21	387	404	414	205	217	225	893	985	1025
10	22	369	374	385	216	224	219	863	945	1085
10	23	416	385	402	218	209	234	882	912	1152
10	24	377	396	368	217	211	215	954	963	1168
10	25	455	399	405	209	218	227	873	954	1125
10	26	416	403	412	205	221	241	861	987	1058
10	27	421	412	410	206	231	238	879	951	1069
10	28	400	400	432	214	205	219	856	1026	1142
10	29	422	412	426	218	208	231	921	986	1025
10	30	431	398	436	205	219	226	865	954	1156
10	31	444	421	357	198	222	198	874	985	1148
10	32	427	401	354	206	222	206	835	945	1196
10	33	435	432	402	210	215	211	855	962	1206
10	34	432	411	369	211	208	215	876	955	1244
10	35	411	386	358	200	224	205	863	1012	1056
10	36	422	424	357	201	219	212	945	1014	1198
10	37	388	391	345	213	232	221	865	965	1122
10	38	399	384	365	206	218	198	902	996	1205
10	39	432	411	365	222	214	206	895	995	1198
10	40	453	442	364	198	200	195	925	981	1104
10	41	432	417	368	201	201	200	965	972	1122
10	42	412	405	397	196	205	212	845	1016	1056
10	43	398	387	402	212	209	221	892	963	1006
10	44	400	376	435	201	200	239	874	1044	1211
10	45	456	431	365	193	205	206	985	964	1252
10	46	431	428	411	213	225	214	888	985	1069
10	47	401	377	384	221	218	198	874	1000	1158

10	48	401	398	402	205	209	221	856	1004	1045
10	49	401	378	355	216	209	191	836	1015	1269
10	50	378	388	400	199	217	228	879	1045	1025
11	1	356	398	433	214	220	230	856	985	1025
11	2	412	401	421	215	218	231	845	988	1125
11	3	389	388	405	219	217	213	869	963	1241
11	4	400	407	426	218	225	216	900	1012	1236
11	5	432	412	401	223	218	218	869	1024	1058
11	6	376	387	403	214	219	217	875	987	1156
11	7	401	399	411	216	221	215	891	957	1258
11	8	387	403	421	216	213	216	886	1013	1036
11	9	421	418	463	196	200	231	854	1045	1145
11	10	418	422	411	201	198	217	874	1009	1205
11	11	416	413	410	220	222	215	865	985	1203
11	12	402	398	396	219	214	218	899	966	1116
11	13	378	399	402	200	212	210	885	1021	1104
11	14	401	416	365	218	209	198	874	1026	1205
11	15	388	391	378	215	210	201	869	1024	1211
11	16	400	426	399	214	211	216	874	1003	1122
11	17	403	396	402	225	234	218	876	987	1058
11	18	387	395	411	222	231	214	853	945	1163
11	19	384	401	399	216	219	214	892	963	1203
11	20	371	387	387	219	222	200	845	954	1240
11	21	400	407	369	219	225	198	832	954	1120
11	22	410	412	354	214	218	215	854	1006	1153
11	23	401	398	321	209	210	211	912	1087	1048
11	24	412	405	425	211	213	254	872	1045	1163
11	25	401	398	400	235	228	235	863	1065	1205
11	26	388	411	403	222	224	243	777	1024	1058
11	27	387	398	369	205	212	195	853	1231	1163
11	28	376	379	401	207	209	213	854	1021	1128
11	29	456	476	444	200	207	251	865	965	1256
11	30	400	399	402	200	205	212	956	945	1049
11	31	413	414	368	198	200	199	832	966	1163
11	32	423	423	414	207	208	218	854	985	1158
11	33	421	411	403	215	216	216	875	945	1059
11	34	392	397	422	206	210	212	864	968	1219
11	35	400	400	396	213	215	210	878	1014	1098
11	36	398	395	402	221	219	200	845	965	1126
11	37	376	381	396	218	209	214	879	999	1522
11	38	345	336	371	205	215	206	863	902	1045
11	39	412	381	385	214	209	212	899	985	1256
11	40	409	412	406	214	221	206	902	964	1145
11	41	413	399	354	206	218	201	845	966	1189
11	42	419	406	387	200	221	216	874	912	1096
11	43	398	356	396	205	208	211	889	1014	1195
11	44	387	401	366	219	224	216	868	1009	1205
11	45	411	421	413	206	215	225	869	921	1187
11	46	400	395	418	215	217	218	891	962	1205
11	47	398	402	325	219	225	222	900	945	1188

11	48	423	411	385	221	232	216	856	956	1245
11	49	398	384	379	209	215	217	845	985	1147
11	50	400	396	409	217	209	213	879	997	1056
12	1	398	378	412	209	215	225	805	956	1435
12	2	376	391	425	212	213	231	865	987	1406
12	3	401	399	388	208	210	198	902	941	1432
12	4	398	378	368	211	208	190	874	963	1396
12	5	388	391	374	218	222	198	865	952	1356
12	6	401	412	385	214	221	212	879	964	1336
12	7	400	389	379	216	217	209	854	954	1432
12	8	392	387	369	221	231	214	878	962	1465
12	9	378	369	395	219	219	201	865	1015	1324
12	10	367	401	391	219	208	200	891	963	1256
12	11	382	399	354	218	215	199	863	1120	1356
12	12	400	386	365	214	198	213	854	957	1456
12	13	399	403	378	215	211	222	874	985	1319
12	14	401	421	396	217	215	219	862	945	1345
12	15	399	403	356	213	207	213	856	912	1298
12	16	387	389	387	219	199	190	854	962	1456
12	17	398	403	377	221	219	201	854	945	1308
12	18	387	399	406	193	216	215	863	978	1452
12	19	423	411	405	209	221	212	875	931	1432
12	20	368	387	395	211	213	201	845	965	1407
12	21	421	409	400	200	231	213	789	1026	1356
12	22	413	411	396	222	219	216	856	978	1243
12	23	381	396	358	198	205	195	854	1000	1345
12	24	400	387	369	205	235	219	863	963	1378
12	25	381	392	388	212	218	190	902	965	1234
12	26	398	400	402	214	210	216	845	965	1421
12	27	412	409	411	200	233	213	900	985	1544
12	28	400	378	425	197	208	228	865	988	1422
12	29	411	395	400	201	198	215	841	999	1345
12	30	434	414	400	198	215	218	882	954	1321
12	31	412	398	415	204	205	231	892	1026	1385
12	32	367	376	396	198	222	213	902	965	1205
12	33	408	421	388	201	218	219	893	1021	1300
12	34	411	388	365	211	209	198	915	965	1345
12	35	427	402	384	225	231	191	865	932	1235
12	36	400	385	400	198	218	221	894	1012	1432
12	37	413	432	402	210	221	219	902	965	1466
12	38	415	399	385	203	216	198	945	978	1345
12	39	425	412	382	215	218	213	863	1014	1328
12	40	399	403	418	214	220	222	859	966	1421
12	41	387	400	396	214	214	212	879	984	1567
12	42	400	376	385	219	222	213	896	931	1209
12	43	412	398	365	208	215	205	862	1012	1342
12	44	423	407	414	210	209	213	874	1000	1543
12	45	413	409	397	203	210	212	865	985	1309
12	46	400	387	406	186	215	215	878	965	1399
12	47	431	422	419	204	218	221	902	985	1344

12	48	434	406	395	194	221	200	854	1029	1365
12	49	413	421	435	183	223	231	865	1047	1321
12	50	400	390	408	225	214	212	878	965	1498
13	1	411	402	375	201	218	198	825	986	1206
13	2	372	387	369	194	203	199	891	965	1199
13	3	387	404	356	214	221	201	878	945	1165
13	4	409	398	387	219	224	191	856	932	1187
13	5	422	416	346	231	254	225	864	945	1156
13	6	399	403	436	199	227	222	879	1002	1205
13	7	406	412	396	214	218	213	895	965	1156
13	8	389	378	425	198	206	215	888	986	1358
13	9	403	400	388	206	212	191	905	996	1206
13	10	421	412	406	217	222	216	952	994	1287
13	11	413	398	356	229	208	198	902	1026	1098
13	12	408	401	406	215	215	213	859	996	1152
13	13	400	378	398	209	225	215	865	945	1069
13	14	405	387	345	214	232	213	891	961	1154
13	15	398	400	387	204	211	197	875	982	1069
13	16	412	408	415	213	210	225	865	985	1174
13	17	422	431	426	208	205	231	888	1006	1202
13	18	401	398	400	219	211	224	879	1021	1196
13	19	422	406	413	203	221	229	847	9658	1206
13	20	409	374	421	224	214	226	852	954	1185
13	21	406	391	400	227	231	219	865	962	1204
13	22	411	382	393	214	221	198	846	952	1362
13	23	401	395	369	217	210	205	912	954	1258
13	24	431	402	400	229	209	213	856	912	1209
13	25	391	384	398	214	208	201	845	956	1154
13	26	387	412	402	206	214	201	874	932	1163
13	27	400	399	400	214	221	198	965	1025	1205
13	28	388	392	391	222	218	209	901	965	1189
13	29	376	387	384	208	209	198	896	982	1278
13	30	408	412	389	204	235	196	956	993	1198
13	31	413	395	377	206	232	213	878	985	1144
13	32	421	398	349	215	241	226	891	976	1098
13	33	427	436	352	209	239	215	856	964	1100
13	34	392	377	368	204	210	209	945	1025	1206
13	35	389	391	409	213	214	221	963	896	1232
13	36	387	376	416	224	198	219	878	855	1152
13	37	400	388	422	213	205	216	902	893	1196
13	38	388	401	419	208	200	215	894	965	1148
13	39	411	381	400	198	200	213	856	1014	1205
13	40	398	400	389	218	222	221	845	965	1241
13	41	431	406	415	231	224	212	879	985	1198
13	42	405	412	413	206	214	222	866	945	1109
13	43	402	398	402	215	218	200	874	963	1204
13	44	396	400	396	219	209	195	956	1052	1144
13	45	412	412	365	220	218	212	897	965	1058
13	46	398	388	387	214	217	190	881	978	1147
13	47	398	400	354	218	224	218	862	996	1098

13	48	387	388	399	229	231	217	945	1036	1269
13	49	376	376	402	196	205	212	863	965	1208
13	50	400	391	400	199	224	209	875	961	1100
14	1	374	387	415	215	207	212	885	941	1145
14	2	398	401	426	206	212	226	896	965	1056
14	3	400	365	385	213	208	198	854	987	1125
14	4	398	400	387	218	222	191	875	985	1069
14	5	400	385	406	214	217	215	845	952	1252
14	6	416	412	416	209	202	209	8789	963	1278
14	7	398	325	385	204	215	194	832	945	1069
14	8	411	365	368	215	217	205	902	852	1241
14	9	432	416	394	207	210	214	890	996	1165
14	10	428	422	416	199	205	223	886	985	1189
14	11	400	378	400	215	221	200	891	945	1175
14	12	421	409	410	214	216	218	856	972	1146
14	13	456	436	413	221	222	212	854	965	1125
14	14	421	421	422	208	215	224	857	948	1205
14	15	398	402	412	218	217	223	863	1006	1277
14	16	378	369	398	205	206	212	852	958	1296
14	17	396	387	368	206	212	198	896	965	1156
14	18	387	402	400	207	209	200	845	921	1036
14	19	378	396	399	198	215	211	902	894	1254
14	20	396	403	368	200	213	205	911	935	1099
14	21	407	354	479	185	200	246	785	856	1211
14	22	413	403	388	231	245	201	865	986	1188
14	23	401	369	425	198	216	226	852	954	1216
14	24	421	478	452	196	225	238	854	962	1248
14	25	423	354	354	216	235	198	875	1002	1125
14	26	404	400	431	214	227	228	845	1054	1098
14	27	419	400	402	216	226	216	900	1065	1201
14	28	406	406	412	219	221	214	852	985	1144
14	29	405	412	400	217	216	221	845	954	1026
14	30	378	386	396	219	222	195	876	1065	1145
14	31	345	369	402	217	210	216	887	1058	1196
14	32	387	409	425	219	224	228	902	985	1205
14	33	398	354	436	221	236	227	856	965	1189
14	34	387	391	400	219	209	198	845	978	1205
14	35	391	402	419	215	204	223	965	1026	1121
14	36	400	387	439	223	231	239	874	954	1056
14	37	432	412	402	209	243	225	856	963	1189
14	38	421	423	419	208	215	227	863	985	1154
14	39	433	432	425	198	209	220	878	905	1045
14	40	401	368	402	206	210	216	902	956	1126
14	41	427	425	418	212	203	224	954	964	1056
14	42	395	400	425	214	218	229	874	988	1205
14	43	401	395	388	209	207	195	872	1012	1285
14	44	389	411	409	215	212	220	862	985	1154
14	45	417	425	458	201	205	237	954	1065	1201
14	46	385	358	436	204	224	224	876	999	1223
14	47	421	416	389	196	215	212	888	978	1208

14	48	395	406	415	203	217	222	796	963	1263
14	49	401	365	428	192	209	221	956	1058	1130
14	50	421	421	429	204	210	228	900	1155	1096
15	1	401	389	385	212	217	192	955	1002	1236
15	2	405	401	396	209	210	200	956	985	1098
15	3	412	367	345	217	206	213	854	1124	1154
15	4	404	345	369	216	204	218	954	956	1122
15	5	399	401	387	201	218	199	879	954	1152
15	6	422	412	354	228	217	213	863	963	1089
15	7	387	368	369	212	215	216	952	957	1136
15	8	401	389	421	204	200	228	896	953	1205
15	9	399	354	403	206	200	224	889	956	1188
15	10	387	386	399	215	200	201	882	947	1098
15	11	396	400	385	219	215	213	845	921	1125
15	12	411	402	378	220	214	200	879	985	1205
15	13	412	422	369	214	221	209	863	962	1263
15	14	409	436	406	218	218	224	856	978	1145
15	15	399	352	418	217	223	226	895	952	1189
15	16	416	412	358	219	214	217	892	963	1250
15	17	403	387	452	219	219	241	845	954	1036
15	18	398	401	432	216	222	229	864	987	1125
15	19	387	395	419	209	210	221	887	952	1098
15	20	413	403	400	207	219	210	902	911	1100
15	21	467	417	402	214	209	223	856	923	1202
15	22	378	387	412	219	224	209	879	912	1189
15	23	376	389	445	221	219	222	921	945	1145
15	24	388	400	436	203	210	218	954	952	1058
15	25	386	352	436	200	218	226	981	941	1036
15	26	400	391	445	210	225	231	952	963	1121
15	27	400	354	489	217	224	254	863	955	1054
15	28	400	358	378	205	219	221	849	945	1287
15	29	376	381	435	214	208	219	895	912	1201
15	30	347	347	406	215	217	210	902	1021	1189
15	31	391	391	412	218	219	208	945	985	1206
15	32	396	396	411	225	208	204	879	997	1098
15	33	392	392	396	221	215	204	863	996	1187
15	34	407	407	387	219	222	219	892	954	1203
15	35	432	432	386	222	231	215	887	985	1152
15	36	367	367	402	208	210	215	856	961	1058
15	37	389	389	400	215	218	198	873	945	1198
15	38	403	403	425	209	214	221	885	976	1205
15	39	388	388	396	195	200	193	897	952	1254
15	40	376	376	365	196	218	209	906	1016	1096
15	41	393	393	358	219	221	217	914	965	1205
15	42	398	398	401	196	209	201	854	954	1125
15	43	403	403	414	217	227	221	867	968	1056
15	44	412	412	406	191	231	203	888	1026	1125
15	45	400	400	396	216	219	198	885	1015	1204
15	46	387	387	391	214	224	212	874	965	1287
15	47	391	391	400	219	221	213	863	995	1160

15	48	395	395	368	224	231	198	854	1025	1036
15	49	387	387	388	216	208	191	854	986	1155
15	50	400	400	392	191	226	205	903	955	1026
16	1	387	364	371	204	201	209	854	986	1236
16	2	401	398	405	212	215	203	854	962	1205
16	3	334	356	396	209	206	216	879	954	1128
16	4	418	402	412	219	214	215	865	981	1156
16	5	401	398	435	209	225	231	892	956	1198
16	6	421	433	406	232	218	213	846	923	1146
16	7	400	356	401	227	219	198	879	965	1158
16	8	444	402	415	196	216	214	885	954	1123
16	9	387	391	465	214	198	258	845	985	1250
16	10	386	378	425	212	219	216	876	974	1209
16	11	417	385	421	209	189	219	832	982	1254
16	12	400	398	368	221	215	231	845	971	1189
16	13	376	385	402	231	214	212	889	983	1201
16	14	376	382	436	198	216	234	875	958	1212
16	15	398	400	401	201	199	221	865	999	1189
16	16	377	368	396	212	201	195	891	995	1205
16	17	386	375	421	199	221	217	856	891	1211
16	18	401	369	452	189	208	243	845	993	1245
16	19	376	354	436	203	216	231	877	986	1269
16	20	392	453	400	188	203	211	902	994	1125
16	21	412	412	412	200	212	210	856	992	1058
16	22	424	431	423	185	198	221	965	1012	1069
16	23	378	426	419	200	211	215	865	965	1158
16	24	377	406	412	212	218	209	863	948	1204
16	25	391	395	366	205	209	199	894	962	1111
16	26	400	385	395	199	211	198	854	932	1024
16	27	356	368	402	212	216	201	886	945	1154
16	28	392	402	437	203	209	229	852	987	1250
16	29	401	366	400	212	215	198	923	1054	1203
16	30	399	400	396	205	225	212	956	965	1146
16	31	387	400	358	218	221	215	823	987	1189
16	32	413	366	400	214	219	197	856	923	1125
16	33	409	412	406	235	241	202	879	900	1120
16	34	417	425	413	198	237	214	856	1016	1205
16	35	421	432	428	205	244	224	892	985	1207
16	36	388	412	429	214	239	227	900	1152	1289
16	37	406	417	409	222	231	213	856	998	1145
16	38	399	356	396	208	205	196	891	865	1163
16	39	412	385	436	214	208	225	856	874	1158
16	40	377	421	411	217	225	212	887	965	1196
16	41	391	387	400	200	212	197	863	986	1147
16	42	400	406	399	209	211	204	897	945	1152
16	43	377	426	412	211	212	216	856	978	1145
16	44	406	368	424	198	211	217	892	963	1168
16	45	387	391	467	206	209	264	884	954	1205
16	46	408	415	478	196	236	264	879	987	1198
16	47	423	408	423	201	228	221	856	923	1096

16	48	401	387	412	214	207	209	873	945	1205
16	49	406	395	401	202	215	214	895	965	1144
16	50	401	399	419	219	226	216	845	966	1023
17	1	389	375	385	215	209	212	889	985	1122
17	2	401	387	402	203	215	200	856	987	1056
17	3	432	423	412	199	213	215	865	963	1251
17	4	421	436	436	211	205	231	867	954	1148
17	5	388	406	412	216	214	210	879	963	1168
17	6	401	398	400	200	216	198	863	975	1147
17	7	428	432	396	287	205	192	892	971	1165
17	8	345	365	385	279	207	209	902	958	1123
17	9	382	391	378	210	216	219	897	972	1125
17	10	398	395	376	211	219	222	854	964	1165
17	11	400	402	394	214	218	216	863	932	1145
17	12	376	385	358	217	217	205	874	991	1208
17	13	378	381	406	206	222	201	879	986	1254
17	14	402	414	422	208	215	219	863	985	1189
17	15	404	427	412	204	215	214	854	965	1205
17	16	387	400	406	206	241	205	900	945	1215
17	17	400	354	409	208	198	210	854	942	1209
17	18	402	407	425	213	206	221	865	973	1258
17	19	423	412	436	228	200	232	886	982	1145
17	20	398	409	421	222	215	213	863	946	1207
17	21	401	421	409	211	219	200	859	958	1298
17	22	409	412	400	201	210	198	879	1026	1152
17	23	400	387	399	204	214	205	902	956	1258
17	24	388	365	400	205	228	214	893	987	1057
17	25	400	387	475	196	200	266	879	1015	1189
17	26	435	406	452	200	206	235	902	965	1254
17	27	421	411	436	201	212	228	986	987	1231
17	28	387	375	400	214	221	198	857	955	1089
17	29	384	369	425	218	214	218	865	926	1045
17	30	378	392	396	206	210	200	878	934	1147
17	31	387	387	419	201	206	215	892	918	1056
17	32	401	407	436	191	215	229	819	985	1214
17	33	422	395	423	205	219	215	854	906	1189
17	34	412	365	425	222	210	213	875	913	1206
17	35	398	387	400	214	226	222	863	925	1125
17	36	387	354	454	227	200	235	854	1025	1189
17	37	401	408	463	203	235	241	891	965	1197
17	38	424	388	425	199	215	216	865	958	1269
17	39	418	392	436	195	211	228	878	1012	1247
17	40	417	391	441	227	214	239	888	985	1239
17	41	391	352	452	219	209	243	846	965	1205
17	42	388	348	465	217	215	231	892	985	1237
17	43	400	354	438	219	224	219	896	978	1233
17	44	387	391	400	198	200	195	906	985	1184
17	45	387	394	400	215	208	186	859	1013	1205
17	46	392	354	389	213	198	192	861	997	1266
17	47	412	413	396	206	236	201	862	965	1033

17	48	399	401	425	209	238	216	905	945	1045
17	49	400	387	398	214	210	195	912	925	1189
17	50	404	399	400	227	219	196	889	980	1298
18	1	456	422	401	219	242	198	854	952	1250
18	2	404	398	396	216	211	200	852	946	1241
18	3	398	345	394	214	214	206	869	932	1263
18	4	439	387	425	216	227	213	894	978	1198
18	5	401	403	436	205	235	218	878	963	1201
18	6	356	354	406	208	210	201	856	945	1203
18	7	385	381	415	210	208	213	965	912	1208
18	8	432	385	417	213	211	210	845	1019	1245
18	9	445	390	398	222	207	204	825	982	1296
18	10	385	412	377	219	234	212	986	971	1256
18	11	421	354	396	205	198	196	885	986	1287
18	12	435	335	436	206	187	228	898	963	1296
18	13	398	375	458	218	205	227	852	952	1298
18	14	400	369	426	215	182	216	845	941	1253
18	15	403	352	357	201	211	191	889	1028	1247
18	16	400	400	411	202	228	212	985	989	1203
18	17	376	344	435	214	205	219	825	1016	1283
18	18	391	388	456	216	211	228	865	986	1254
18	19	387	354	454	215	204	231	895	965	1269
18	20	392	387	387	213	211	195	875	964	1254
18	21	400	352	413	205	206	205	826	985	1285
18	22	422	369	399	222	200	198	875	932	1296
18	23	406	398	401	211	214	196	892	956	1201
18	24	388	395	397	211	216	209	863	954	1230
18	25	415	378	412	232	213	213	845	963	1232
18	26	423	394	452	216	201	221	876	921	1205
18	27	421	354	431	214	187	218	863	944	1344
18	28	398	369	399	221	178	196	856	935	1367
18	29	413	401	425	201	231	215	893	902	1269
18	30	432	403	413	216	229	209	854	984	1245
18	31	399	388	400	222	196	194	882	1096	1209
18	32	412	411	419	212	211	211	846	902	1256
18	33	422	387	428	211	198	219	892	965	1289
18	34	406	412	422	215	231	213	876	931	1247
18	35	423	415	419	205	224	204	895	925	1250
18	36	435	378	385	213	198	205	875	906	1206
18	37	406	398	400	213	205	201	789	1056	1278
18	38	432	356	377	226	177	212	985	965	1225
18	39	456	345	365	210	198	206	885	1023	1254
18	40	422	378	381	205	196	195	882	1056	1258
18	41	400	369	374	209	203	208	874	1086	1245
18	42	356	395	400	201	215	206	856	996	1265
18	43	376	394	385	205	200	198	842	1006	1248
18	44	400	384	378	196	198	201	785	997	1247
18	45	419	400	402	193	254	196	856	1012	1320
18	46	451	421	418	206	231	209	821	985	1385
18	47	389	396	400	214	222	198	845	965	1250

18	48	403	411	423	219	215	216	875	985	1310
18	49	398	354	398	216	208	212	898	983	1345
18	50	387	400	395	200	224	215	805	978	1325
19	1	427	415	416	192	212	211	845	1007	1325
19	2	403	401	396	201	221	215	865	986	1356
19	3	400	398	389	198	215	209	856	945	1205
19	4	399	378	452	216	205	221	896	896	1321
19	5	421	416	367	225	210	206	832	978	1256
19	6	431	403	429	216	214	216	879	954	1205
19	7	423	395	406	224	211	201	842	921	1398
19	8	400	411	456	217	212	228	899	984	1387
19	9	411	406	397	205	212	195	889	983	1361
19	10	417	398	368	219	203	211	965	976	1250
19	11	412	388	428	220	215	212	865	989	1215
19	12	420	391	436	211	218	215	845	931	1252
19	13	398	387	488	198	214	234	875	954	1289
19	14	400	356	456	196	217	221	893	962	1254
19	15	392	365	398	216	206	196	852	978	1264
19	16	395	401	425	198	219	216	777	1098	1234
19	17	400	398	369	200	206	199	895	945	1262
19	18	412	398	421	215	200	213	879	931	1258
19	19	398	365	406	225	198	196	865	1016	1205
19	20	387	358	369	197	195	213	896	965	1287
19	21	387	346	356	199	210	215	854	1017	1264
19	22	411	402	401	205	201	196	826	1025	1263
19	23	432	368	396	205	213	209	891	963	1264
19	24	428	412	432	217	214	217	900	1047	1225
19	25	423	395	400	224	212	215	856	987	1219
19	26	378	366	378	212	199	201	879	1058	1204
19	27	381	391	400	204	185	199	893	1012	1251
19	28	387	402	412	196	239	203	854	900	1265
19	29	421	406	416	197	242	215	874	954	1267
19	30	400	354	418	200	198	206	859	934	1244
19	31	409	378	423	189	188	212	863	1012	1296
19	32	433	359	428	196	178	221	821	954	1254
19	33	411	378	400	200	200	196	896	968	1223
19	34	398	382	384	189	201	201	889	977	1223
19	35	401	398	402	186	215	213	802	903	1289
19	36	400	366	412	204	211	204	825	954	1254
19	37	387	358	400	216	185	219	847	963	1206
19	38	356	412	416	222	235	201	815	978	1296
19	39	413	365	391	222	174	211	875	932	1287
19	40	422	395	411	234	186	198	865	941	1254
19	41	418	395	400	231	200	216	895	963	1205
19	42	421	354	452	228	178	241	879	957	1298
19	43	400	374	438	212	181	222	893	921	1245
19	44	400	395	401	209	200	186	902	1052	1221
19	45	431	394	396	214	185	222	899	978	1227
19	46	398	402	411	212	201	203	859	951	1298
19	47	402	417	418	216	231	210	867	984	1250

19	48	412	406	412	228	221	204	892	915	1242
19	49	432	381	403	239	198	198	876	974	1265
19	50	437	367	412	232	178	212	877	965	1355
20	1	434	392	402	231	196	215	959	1023	1096
20	2	456	385	395	254	185	217	962	1011	1125
20	3	400	374	425	221	187	216	946	986	1166
20	4	398	365	436	219	212	221	975	998	1204
20	5	387	398	406	205	215	210	964	1012	1025
20	6	402	354	424	236	221	217	879	1016	1156
20	7	398	321	400	196	214	201	862	998	1293
20	8	398	388	406	197	218	212	856	901	1254
20	9	378	396	422	214	219	213	898	1016	1205
20	10	376	387	431	213	205	215	863	986	1240
20	11	401	349	406	218	198	198	895	1024	1277
20	12	399	378	468	223	215	236	845	987	1252
20	13	432	395	435	217	208	215	856	965	1152
20	14	411	365	421	213	254	218	876	985	1369
20	15	422	387	437	214	221	221	859	1023	1364
20	16	402	358	396	201	198	200	845	919	1345
20	17	387	402	388	196	245	198	921	1056	1398
20	18	391	408	388	214	235	221	965	978	1258
20	19	413	406	412	218	241	212	854	963	1356
20	20	423	395	400	222	208	198	878	1012	1254
20	21	431	365	402	205	198	201	889	1015	1324
20	22	422	398	381	216	185	212	896	1005	1325
20	23	395	401	427	219	231	219	936	986	1452
20	24	401	366	386	215	174	195	945	1052	1325
20	25	396	357	412	213	181	213	974	1006	1208
20	26	400	349	402	202	169	201	895	1045	1288
20	27	413	398	456	221	189	231	963	989	1201
20	28	434	351	389	210	196	185	952	1024	1220
20	29	412	369	474	200	200	261	918	971	1263
20	30	442	389	412	233	199	214	994	996	1154
20	31	423	400	421	231	225	216	905	1045	1058
20	32	409	388	436	218	210	218	945	1087	1256
20	33	408	368	412	201	198	199	985	1025	1247
20	34	452	345	436	242	169	231	979	1009	1256
20	35	438	325	447	218	174	231	963	1045	1369
20	36	422	387	415	225	186	216	974	985	1358
20	37	412	395	406	227	198	198	946	1078	1345
20	38	416	397	428	218	189	214	979	1056	1209
20	39	434	388	400	217	187	201	869	1025	1231
20	40	400	378	394	228	173	205	956	1065	1356
20	41	434	391	425	234	185	219	978	1012	1345
20	42	401	378	400	219	189	214	900	1007	1362
20	43	413	390	468	215	185	259	956	989	1315
20	44	432	381	406	213	175	210	947	951	1336
20	45	413	396	437	205	190	228	874	964	1352
20	46	463	398	416	238	195	221	956	1065	1325
20	47	421	379	425	231	165	218	988	1012	1345

20	48	435	387	435	341	191	227	879	1014	1398
20	49	421	354	426	232	161	216	856	1067	1204
20	50	427	389	400	228	188	215	893	1015	1398
